# Increased cytokine production capacity and persistent inflammation after achieving remission of Cushing’s syndrome

**DOI:** 10.1016/j.jcte.2026.100443

**Published:** 2026-05-03

**Authors:** Pepijn van Houten, Annenienke C. van de Ven, Antonius E. van Herwaarden, Mihai G. Netea, Martin Jaeger, Romana T. Netea-Maier

**Affiliations:** a Department of Internal Medicine, Division of Endocrinology, Radboud University Medical Center, Nijmegen, the Netherlands; b Department of Laboratory Medicine, Radboud University Medical Center, Nijmegen, the Netherlands; c Department of Internal Medicine, Radboud Community for Infectious Diseases, Radboud University Medical Center, Nijmegen, the Netherlands; d Department of Metabolism and Immunology, Life and Medical Sciences Institute, University of Bonn, Bonn, Germany; e Research Center for Functional Genomics, Biomedicine and Translation Medicine, Iuliu Hatieganu University of Medicine and Pharmacy, Cluj-Napoca, Romania

**Keywords:** Cushing’s syndrome, Cushing’s disease, Inflammation, Monocytes, Innate immunity

## Abstract

Endogenous Cushing’s syndrome (CS) is characterized by chronic hypercortisolism. After achieving remission, morbidity remains increased and quality of life remains impaired. Moreover, the incidence of inflammatory disorders increases after remission of CS. Despite these immune-mediated clinical consequences, the course of innate immune cell function after remission of CS is poorly known. In this study we aimed to assess changes in innate immune cell function and systemic inflammatory markers after achieving remission of CS. Blood samples were collected from nine CS patients (six adrenal CS and three Cushing’s disease) at diagnosis and after achieving remission and fully tapering off glucocorticoid suppletion. Peripheral blood mononuclear cell (PBMC) cellularity and monocyte-derived cytokine responses upon 24 h of *ex vivo* stimulation were compared between at diagnosis and after achieving remission. Changes in circulating inflammation-related proteins were assessed by proximity extension assay proteomics and ELISA. After achieving remission of CS, the PBMC fraction showed higher percentages of lymphocytes and lower percentages of monocytes. Production of pro-inflammatory cytokines IL-6 and IL-8 by monocytes after 24 h of stimulation with microbial stimuli was higher after achieving remission, while production of anti-inflammatory cytokines IL-10 and IL-1Ra was lower. Proteomic analysis of plasma identified seven inflammation-related proteins with increased expression and eleven proteins with decreased expression after achieving remission. Concentrations of circulating IL-6, IL-8 and CRP did not change after remission. In conclusion, increased pro-inflammatory cytokine production by monocytes and persistent systemic inflammation could partially explain increased morbidity and incidence of inflammatory disorders after achieving remission of CS.

## Highlights

• After achieving remission of hypercortisolism, systemic inflammation persists.
• Meanwhile, proinflammatory cytokine (IL-6 and IL-8) production capacity increases.
• Additionally, the production capacity of IL-1Ra decreases.
• This explains the increased incidence of inflammatory disorders in these patients.

## Graphical abstract

**Figure:**
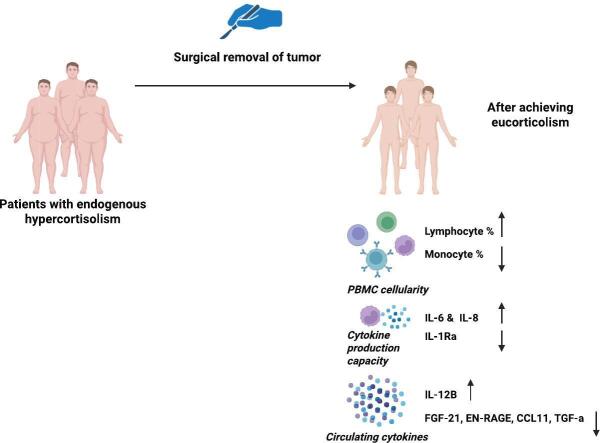

## Introduction

Endogenous hypercortisolism leads to signs and symptoms that constitute Cushing’s syndrome (CS). Endogenous CS is most commonly caused by an corticotropin (ACTH)-producing adenoma of the anterior pituitary gland, in which case it is called Cushing’s disease (CD) (1). Other causes of CS are cortisol-secreting adrenal tumors, adrenal hyperplasia or ACTH-producing ectopic tumors. Treatment of CS consists of removing the causing tumor, by transsphenoidal adenomectomy or by adrenalectomy. For CD, recurrences are prevalent, with recurrence rates after curative pituitary surgery up to 35% of patients (2).

CS can cause a broad variety of signs and symptoms and is associated with significantly increased morbidity and mortality, among which cardiovascular events, venous thromboembolism, increased infection incidence and neuropsychiatric symptoms, all leading to reduced quality of life [1], [3], [4]. Although achieving remission of CS results in the resolution of many symptoms, quality of life often remains impaired even after patients with CS achieve remission, partly because of glucocorticoid withdrawal syndrome [5], [6], [7]. Moreover, mortality rates remain increased in patients with CS even after they achieve remission (8). An interesting phenomenon is the apparent rebound autoimmunity after achieving remission of CS. Several studies have shown an increased incidence of various inflammatory disorders after achieving remission of CS, such as thyroiditis, sarcoidosis and rheumatoid arthritis [9], [10], [11], [12], [13]. An imbalance between subsets of T-lymphocytes and altered thymocyte development have been suggested as possible causes of this phenomenon, but the exact mechanism, and especially the role of the innate immune system are not yet understood [3], [14].

Several studies have shown that circulating concentrations of inflammatory biomarkers such as C-reactive protein (CRP), interleukin-6 (IL-6), IL-8 and tumor necrosis factor (TNF) are elevated in patients with active CS compared to healthy controls [15], [16], [17], [18], [19], [20], [21]. A previous study from our group confirmed these findings (22). In addition, we showed that the peripheral blood mononuclear cell (PBMC) fraction of patients with CS consists of more monocytes and less lymphocytes, compared to the PBMC fraction of healthy controls. Furthermore, monocytes of CS patients produced less proinflammatory cytokines, specifically IL-1β, IL-6 and TNF, upon *ex vivo* stimulation, which potentially plays a role in the increased incidence of infections in patients with active CS. Data on the dynamics and reversibility of these changes upon treatment of hypercortisolism are scarce. As such, Shah *et al*. and Barahona *et al.* showed ongoing elevated markers of systemic inflammation in patients after achieving remission of CS, when compared to healthy controls [15], [16]. In addition, Vogel *et al*. showed persisting levels of low-grade inflammation during the glucocorticoid withdrawal phase after treatment of CS (23). This could, partially, explain the remaining increased cardiovascular risk. Whether the immune cell phenotype also remains affected after achieving remission of CS has not yet been investigated.

In the current study, the course of immune cell phenotype and systemic inflammatory markers after remission of CS were assessed prospectively in a subset of a cohort of CS patients that was previously described cross-sectionally during the active phase of the disease (22). Given the strong effects of the innate immune responses in the pathogenesis of numerous comorbidities associated with CS, insights from this study may serve as important starting points in understanding the pathogenesis of long-term complications of CS such as infections, autoimmune disorders and cardiovascular complications.

## Materials and methods

### Subjects and study procedures

All consecutive adult patients diagnosed with CS and presenting at the Radboud University Medical Center in Nijmegen, the Netherlands, between August 2020 and March 2022 who fulfilled the inclusion criteria (active treatment naïve CS and aged ≥ 18 years) were invited to participate in the study. Patients were diagnosed according to the guidelines (24). Exclusion criteria were: inflammatory or infectious comorbidities, active malignancies, use of statins, use of systemic immunosuppressive medication, inadequately treated hypertension (defined as systolic blood pressure ≥ 160 mmHg or diastolic blood pressure ≥ 100 mmHg), inadequately controlled diabetes mellitus (defined as HbA1c concentration of > 69 mmol/mol in the last year), recent ischemic cardiovascular disease, pregnancy and a self-reported alcohol intake of > 21 units per week. The baseline characteristics including innate immune cell function and biomarkers of systemic inflammation were compared between CS patients in the active phase of disease and healthy controls and reported previously (22).

All patients underwent surgery, either transsphenoidal adenomectomy for CD or adrenalectomy for adrenal CS and all patients received peri- and postoperative glucocorticoid supplementation as stress dosage. Adrenal function was initially evaluated with a postoperative morning fasting cortisol concentration. If the cortisol concentration was < 200 nmol/L, glucocorticoid replacement therapy was continued. Afterwards, the dose was tapered off according to symptoms and fasting cortisol concentrations. Complete recovery of adrenal function as assessed by spontaneous fasting cortisol concentrations or a 250 µg ACTH stimulation test after discontinuation of glucocorticoid supplementation. All patients included in the baseline study who also had documented remission after the primary treatment and who successfully tapered off glucocorticoid supplementation were invited to donate a second blood sample to assess longitudinally the reversibility of the changes in the innate immune function and markers of systemic inflammation upon remission of CS. Patients that developed exclusion criteria after the primary treatment were excluded from the post remission blood drawing. Remission of CS after treatment was defined as either a morning cortisol concentration of ≤ 50 nmol/L or an adequate cortisol suppression after a 1 mg dexamethasone suppression test or a late-night salivary cortisol concentration within the reference range.

Blood was drawn in ethylenediaminetetraacetic acid (EDTA) tubes at diagnosis before the start of cortisol-lowering medication, and after achieving remission of CS after tapering off glucocorticoid replacement therapy. To minimize the effect of diurnal rhythm, blood was always drawn in the morning.

The study was approved by the local institutional review board (dossier number 2020–6440). Procedures were conducted according to the principles of the Declaration of Helsinki. Informed consent was obtained from all participants before study procedures.

### Plasma hormonal assays

Separated plasma from EDTA tubes was stored at −80°C until analysis. Cortisol and its precursor 11-deoxycortisol measured in plasma by liquid chromatography-tandem mass spectrometry (LCMSMS) as described elsewhere (25). Sample preparation was performed by protein precipitation and subsequent solid phase extraction. None of the participants used oral contraceptives or other drugs that up- or downregulate the concentrations of cortisol-binding globulin and thus interfere with this assay.

### PBMC isolation

Peripheral blood mononuclear cells (PBMCs) were isolated from EDTA whole blood by gradient density centrifugation using Ficoll-Paque PLUS (GE Healthcare). Cell counts of subpopulations in the PBMC fraction were analyzed by a Sysmex XN-450 hematology analyzer (Sysmex Corporation).

### PMBC stimulations

Stimulation experiments were performed in round-bottom 96-well plates using Roswell Park Memorial Institute (RPMI) medium (Gibco), supplemented with gentamycin 50 µg/mL, pyruvate 1 mM and glutamax 2 mM. Per well, 5x10^5^ PBMCs were stimulated in duplicate. PBMCs were stimulated for 24 h to assess monocyte-derived cytokine production and for 7 days to assess lymphocyte-derived cytokine production. 24 h stimulations were performed with purified *E. Coli* derived lipopolysaccharide (LPS, serotype O55:B5, Sigma-Aldrich, 1 ng/mL and 10 ng/mL), *Candida albicans (C. albicans*, UC 820, 1x10^6^/mL), *Staphylococcus aureus (S. aureus,* Rosenbach ATCC 25923, 1x10^6^/mL) or RPMI as medium control. 7 days experiments were performed in medium supplemented with 10% human pooled serum, with stimuli *C. albicans* (1x10^6^/mL), *S. aureus* (1x10^6^/mL) or RPMI as medium control. After incubation at 37°C, 5% CO_2_, supernatants were collected and stored at −20°C until measurement. *C. albicans* and *S. aureus* were grown overnight at 37°C in Sabouraud and Brain Heart Infusion broth, respectively. Microorganisms were harvested by centrifugation, washed twice in PBS, and resuspended. *C. albicans* yeasts were heat-killed for 30 min at 95°C.

### ELISA

Cytokine production after stimulation was measured at the end of the trial by enzyme-linked immunosorbent assay (ELISA) using commercial DuoSet ELISA kits (R&D Systems) in accordance to the manufacturer’s protocols. TNF, IL-1β, IL-6, IL-8, IL-10 and IL-1 receptor antagonist (IL-1Ra) were measured in supernatants from the 24 h stimulated PBMCs. IL-17, IL-22 and IFN-γ were measured in supernatants from the 7 days stimulated PBMCs. CRP was measured in EDTA plasma using a human CRP DuoSet ELISA kit (R&D Systems). Three external controls were included on each plate in the assay.

### Proximity extension analysis (PEA)

Inflammation-related proteins were measured in EDTA plasma using the commercial Target 96 Inflammation panel of Olink proteomics. 92 proteins were measured by multiplex proximity extension assay, as quantified by real-time PCR.

### Statistical analysis

Data were analyzed using GraphPad Prism (version 8.02; GraphPad Software). Baseline characteristics are described as part of whole or as median with range. Cell counts, steroid hormone concentrations and cytokine concentrations were compared using the non-parametric Wilcoxon signed-rank test. In order to correctly assess the cytokine production capacity, taking into account the changes in PBMC composition, monocyte-derived cytokine production after 24 h of stimulation was normalized by dividing the cytokine concentration (in pg/mL) by monocyte counts (as per 1x10^5^ monocytes).

PEA results were analyzed using R (version 4.2.2), with packages “ggbiplot” and “ggplot2”. A protein was included in the analysis only if ≥ 75% of samples were above limit of detection. Normalized protein expression (NPX) values were expressed on a log2 scale and were linearized for fold-change calculations. Proteins were compared between diagnosis and after remission by Wilcoxon signed-rank test.

All statistical analyses were performed two-tailed and *p* < 0.05 was considered statistically significant.

## Results

### Patient characteristics

**Fig. 1:**
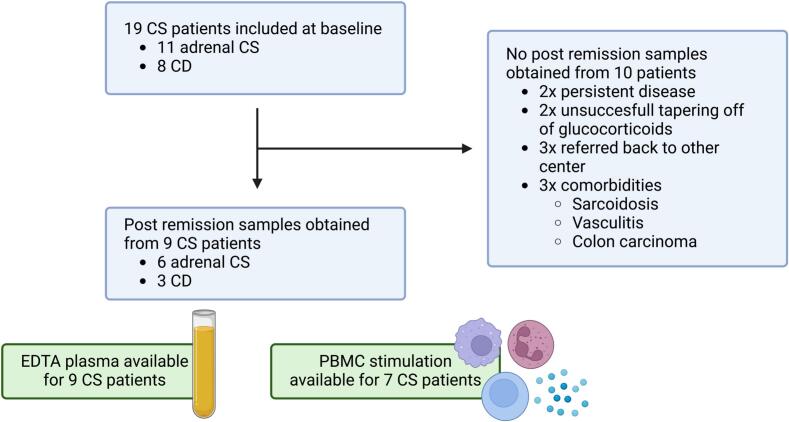
**Inclusions procedures and reasons for not obtaining post remission samples.** CS: Cushing’s syndrome; CD: Cushing’s disease; PBMC: peripheral blood mononuclear cells.

Post remission samples were obtained in nine out of 19 patients with CS that were included at baseline. The procedure of inclusion and reasons for not obtaining post remission samples are depicted in Fig. 1. Three patients had CD and six patients had adrenal CS. Median age was 47 years at diagnosis (range: 31–64 years) and eight out of nine patients were women. Four patients were treated with cortisol-lowering medication (metyrapone) preoperatively. All nine patients achieved remission after surgery. Immunohistochemical staining confirmed an ACTH positive adenoma for all patients with CD and a cortical adenoma for all patients with adrenal CS. Median time between baseline and post remission sample and between surgery and post remission sample were respectively 18 months (range: 13–27 months) and 14 months (range: 11–26 months). The median time between starting and tapering off of hydrocortisone supplementation was 10 months (range: 1–20 months). Median BMI was lower at the time of post remission blood drawing (26.6 kg/m^2^, range: 21.7–31.5) than at baseline (30.1 kg/m^2^, range: 24.7–33.8).

### After achieving remission, percentages of lymphocytes increase and monocytes decrease

**Fig. 2:**
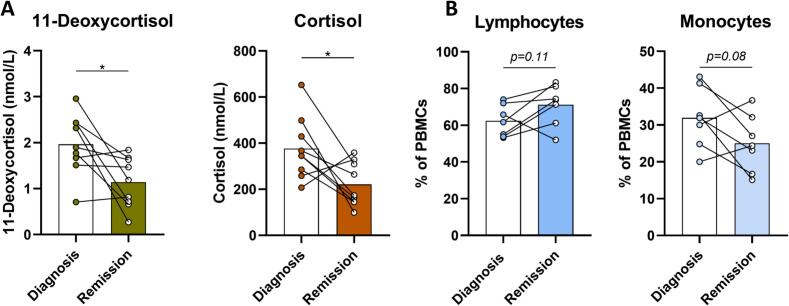
**Changes in plasma glucocorticoids and white blood cells subpopulations after patients with Cushing’s syndrome achieve remission. (A)** Plasma concentrations of steroid hormones 11-deoxycortisol and cortisol at diagnosis and after achieving remission. **(B)** Percentages of lymphocytes and monocytes of the peripheral blood mononuclear cell (PBMC) fraction. * indicates *p* < 0.05.

Steroid hormones cortisol and 11-deoxycortisol were measured by LCMSMS in EDTA plasma. After achieving remission, the concentrations of these hormones were significantly lower compared to concentrations measured at diagnosis (Fig. 2A). Percentages of lymphocytes and monocytes of the PBMC fraction were compared between diagnosis and after achieving remission. Although not statistically significant, the percentage of lymphocytes increased and the percentage of monocytes decreased after achieving remission (Fig. 2B).

### Increased production of IL-8 and IL-6 after remission of CS

**Fig. 3:**
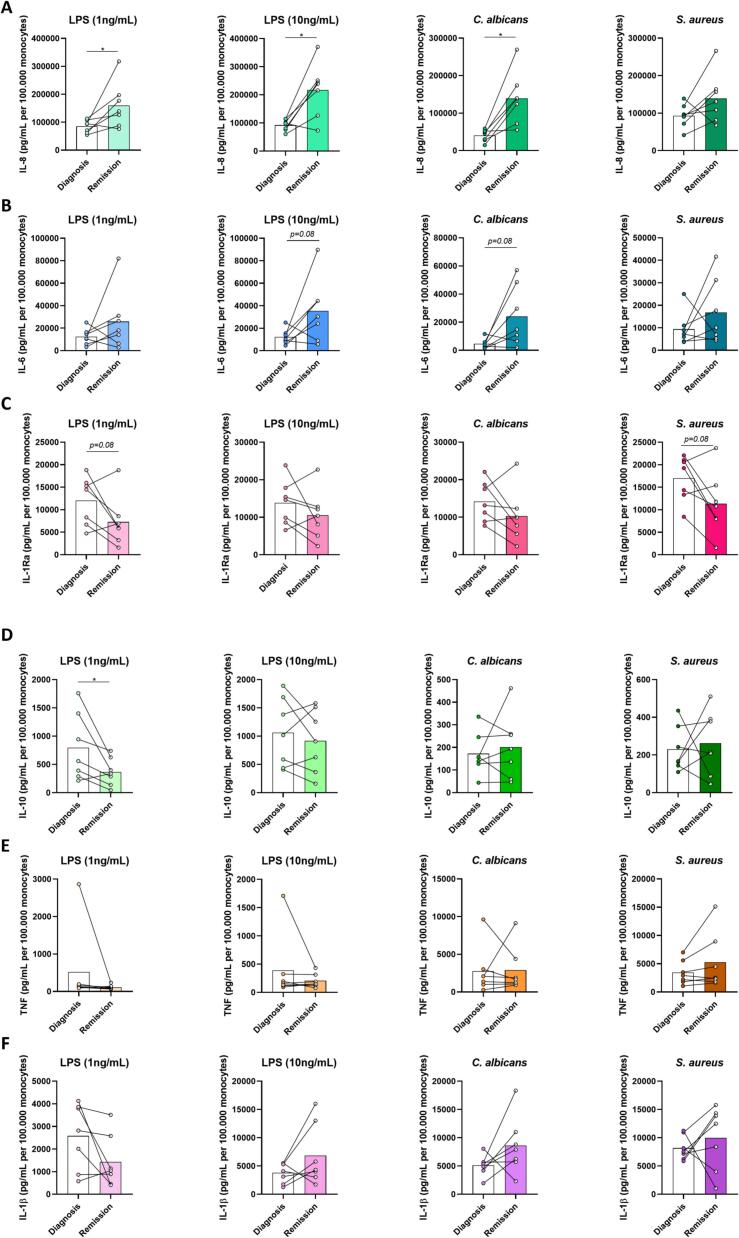
**Increased proinflammatory and decreased antiinflammatory cytokine production capacity in monocytes from Cushing’s patients after achieving remission**. Concentrations of cytokines IL-8 **(A)**, IL-6 **(B)**, IL-1Ra **(C)**, IL-10 **(D)**, TNF **(E)** and IL-1β **(F)** after 24 h of PBMC stimulation at diagnosis and after achieving remission, all corrected for the percentage of monocytes. * indicates *p* < 0.05.

Monocyte-derived cytokines were measured in supernatants from 24 h stimulated PBMCs that were obtained at baseline and after achieving remission and tapering off glucocorticoid replacement therapy (Fig. 3). Because percentages of monocytes within the PBMC fraction significantly changed after achieving remission from CS, the cytokine production per 1.0x10^5^ monocytes was calculated as well. This way, cytokine production capacity of stimulated PBMCs could be compared between baseline and after achieving remission. PBMCs produced significantly more IL-8 after stimulation after achieving remission from CS (Fig. 3A). The same trend could be observed for IL-6, however this was not statistically significant (Fig. 3B). In contrast, for anti-inflammatory cytokines IL-1Ra and IL-10, a trend towards lower production after achieving remission could be observed (Fig. 3C & D). For production of IL-1β and TNF, no trend could be observed comparing baseline and post remission samples (Fig. 3E & F).

**Fig. 4:**
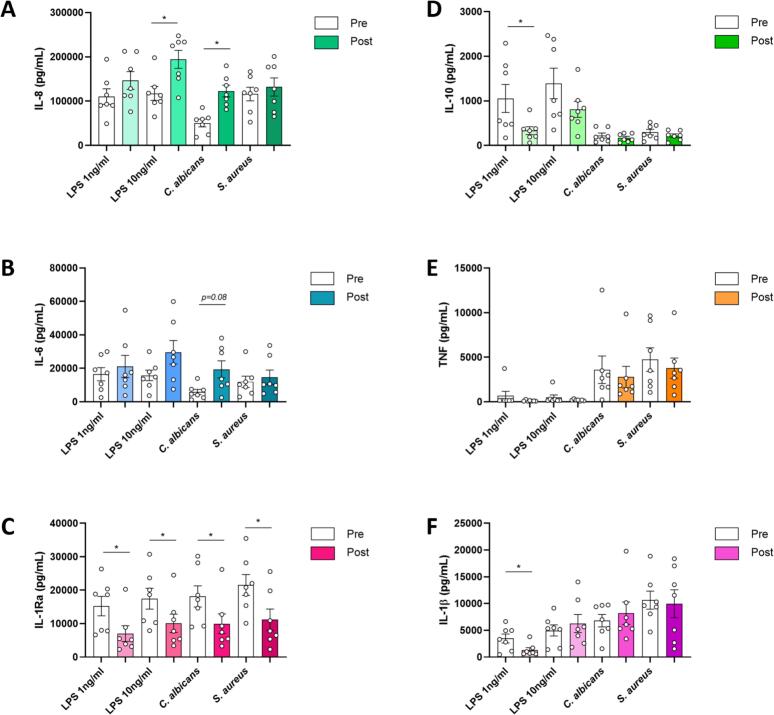
**Cytokine production capacity in PBMCs without correction for monocyte percentages.** Concentrations of cytokines IL-8 **(A)**, IL-6 **(B)**, IL-1Ra **(C)**, IL-10 **(D)**, TNF **(E)** and IL-1β **(F)** after 24 h of PBMC stimulation at diagnosis and after achieving remission, not corrected for percentages of monocytes. * indicates *p* < 0.05.

The non-corrected cytokine concentrations, which more accurately represent the cumulative *in vivo* effects, are shown in Fig. 4. Like in the monocyte-percentage corrected analysis, concentrations of IL-8 and IL-6 were higher after achieving remission and concentrations of IL-1Ra were lower after achieving remission of CS. For IL-10, production capacity was lower after achieving remission for stimulations with LPS 1 ng/mL, but not for the other stimulations. In addition, the production capacity for IL-1β was lower after stimulation with LPS 1 ng/mL, but not for the other stimulations. For TNF, no differences were observed in production capacity at diagnosis and after achieving remission.

**Fig. 5:**
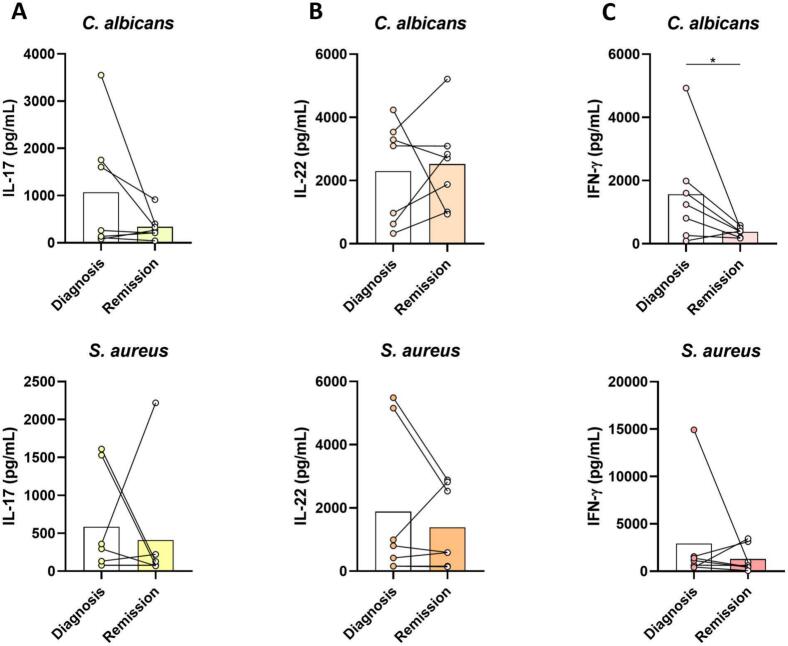
**Lymphocyte derived cytokines before and after achieving remission of Cushing’s syndrome.** Concentrations of IL-17 **(A)**, IL-22 (**B)** and IFN-γ **(C)** after 7 days of PBMC stimulation. * indicates *p* < 0.05.

Lymphocyte-derived cytokines were measured in supernatants from 7 days stimulated PBMCs (Fig. 5). IFN-γ production was lower after achieving remission. This was statistically significant for stimulation with *C. albicans*, but not for stimulation with *S. aureus*. For production of IL-17 and IL-22, no such trend could be observed.

### Circulating inflammatory proteome after remission of CS

**Fig. 6:**
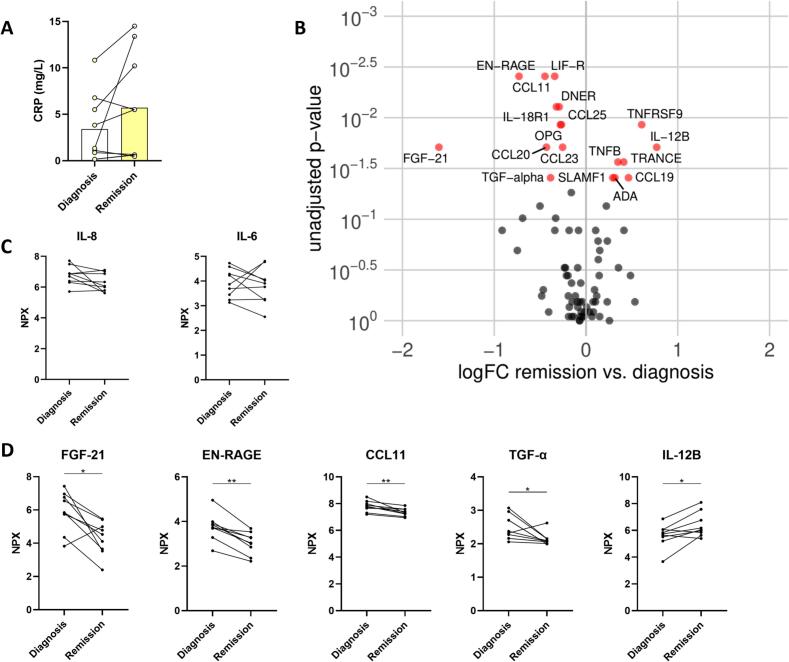
**Changes in de inflammatory plasma proteome after achieving remission of Cushing’s syndrome. (A)** CRP concentrations in EDTA plasma at diagnosis and after achieving remission. **(B)** Vulcano plot showing increased and decreased inflammation-related proteins comparing proteomics in patients after remission and at diagnosis. **(C)** NPX values of IL-8 and IL-6. **(D)** NPX values of FGF-21, EN-RAGE, CCL11, TGF-α and IL-12B. NPX: normalized protein expression; FC: fold change. * indicates p < 0.05 ** indicates p < 0.01.

Circulating inflammatory biomarkers were measured in EDTA plasma. CRP concentrations showed large interindividual variation. No trend could be observed comparing concentrations at diagnosis and after achieving remission (Fig. 6A).

Seventy-seven of 92 inflammation-related proteins could be included in the PEA analysis. The other 15 proteins were not detectable in > 25% of samples. Fig. 6B shows a Vulcano plot comparing the proteome after achieving remission to the proteome at diagnosis. Seven proteins were higher after achieving remission than at diagnosis (SLAMF1, ADA, TNFB, TRANCE, CCL19, IL-12B and TNFRSF9), while eleven proteins were lower after achieving remission (CCL11, CCL20, CCL23, CCL25, OPG, TGF-α, IL-18R1, LIF-R, EN-RAGE, DNER and FGF-21). NPX values of cytokines IL-8 and IL-6 were not significantly different after achieving remission (Fig. 6C). Proteins FGF-21, EN-RAGE, CCL11 and TGF-α were among the proteins that were higher in the comparison between CS patients and healthy controls, while IL-12B was among the proteins that were lower in CS patients (22). Fig. 6D shows the trend of these proteins after achieving remission.

## Discussion

Our prospective observational study shows that after achieving remission of CS, *ex vivo* proinflammatory cytokine production capacity of monocytes increases. Moreover, production capacity of anti-inflammatory cytokines decreases. This novel finding is essential because unlike the basal levels of the circulating inflammatory proteome it provides a better understanding of the responsiveness of the immune system in pathologic conditions and is indicative of how effective the immune system can respond against pathogens as well as other endogenous and exogenous stimuli. This is crucial in the context of infections, autoinflammatory and autoimmune diseases and cancer, and affects metabolism and aging processes [26], [27]. In addition, percentages of lymphocytes and monocytes within the PBMC fraction increase and decrease respectively. Furthermore, we observed noticeable changes in the circulating inflammatory proteome, although concentrations of several biomarkers of systemic inflammation commonly used such as CRP, IL-6 and IL-8 did not change after achieving remission of CS.

After achieving remission of CS, the percentage of lymphocytes within the PBMC fraction increased while the percentage of monocytes decreased. Interestingly, in a previous study investigating the baseline immune characteristics of this cohort, the percentage of lymphocytes and monocytes of CS patients were respectively lower and higher than that of healthy controls (22). Thus, after remission of CS, percentages of these cell populations shift towards the physiological situation. The lymphocyte-to-monocyte ratio (LMR) is a general marker that is negatively correlated to systemic inflammation and a lower LMR has been associated with amongst others metabolic syndrome (28). The shift in cell percentages as observed in the present study could thus be indicative of resolution of metabolic syndrome and decrease in systemic inflammation after achieving remission of CS. It would be tempting to extrapolate these findings on changes in immune cell populations to hematopoietic progenitors. However, in the current study no bone marrow was available. Future studies could investigate the progenitor cell subpopulations in active and cured CS to further assess the effects of hypercortisolism on hematopoiesis.

In addition, we observed higher *ex vivo* production of proinflammatory cytokines IL-6 and IL-8 and lower production of anti-inflammatory cytokines IL-1Ra and IL-10 after achieving remission of CS. These differences were observed both with and without correcting for the percentage of monocytes, so both at cellular and cumulative levels respectively. In the previous study where we assessed monocyte production capacity at CS diagnosis, monocytes of CS patients showed lower *ex vivo* proinflammatory cytokine production capacity compared to healthy controls (22). Patients with active CS have an elevated infection risk, and the increased production capacity of proinflammatory cytokines could be an explanation for a decrease in this risk after remission of CS (3). On the other hand, an increased production capacity of proinflammatory cytokines has also been associated with an increased risk of inflammatory diseases. Production of IL-6 and IL-8 by PBMCs are for example also elevated in patients with psoriasis, systemic lupus erythematosus and rheumatoid arthritis [29], [30], [31]. Therefore, the higher production capacity of proinflammatory cytokines after recovery from hypercortisolism in our patient series could also explain the high incidence of inflammatory diseases that is observed in CS patients after achieving remission [9], [10], [11], [12], [13]. Future studies on how remission of hypercortisolism mechanistically results in this elevated cytokine production capacity are warranted as they could lead to the discovery of new therapeutic targets or biomarkers for inflammatory diseases.

Next, we assessed the inflammatory proteome and identified seven inflammation-related proteins with increasing and eleven proteins with decreasing plasma levels after achieving remission of CS. Interestingly, FGF-21, EN-RAGE, CCL11 and TGF-α were among the proteins that decreased after remission of CS, while these proteins were higher in patients with active CS compared to healthy controls in the previous baseline study (22). The opposite was observed for IL-12b, which was lower in patients with active CS compared to healthy controls, while plasma levels increased after remission of CS. Interestingly, in a recent proteomic study FGF21 and CCL11 were among the proteins that were higher expressed in participants with obesity and metabolic syndrome compared to participants with obesity and no metabolic syndrome (32).

FGF-21 (fibroblast growth factor 21) is a hormone that is primarily produced by hepatocytes and is involved in regulating energy balance and glucose homeostasis. Interestingly, a previous study showed that glucocorticoids induce Fgf21 expression in hepatocytes, both *in vivo* and *in vitro* (33). Furthermore, studies have shown that circulating FGF-21 concentrations are higher in patients with type 2 diabetes mellitus than in healthy controls [34], [35]. Both lower glucocorticoids and better glycemic control could explain the decrease in FGF-21 after achieving remission of CS.

IL-12b is the subunit of IL-12, an interleukin that is primarily produced by antigen presenting cells like dendritic cells and macrophages and induces cytotoxic activity of T-cells and natural killer cells. IL-12 has been found to be increased in several autoinflammatory diseases (36). Therefore, given that both concentrations of IL-12b and incidence of autoinflammatory disease increase after achieving remission of CS, the question remains whether these associations are related pathogenetically.

Interestingly, the concentration of the more commonly used inflammatory biomarkers CRP, IL-6 and IL-8 did not change after achieving remission of CS, while IL-6 and IL-8 were higher in patients with active CS compared to healthy controls in the previous baseline study (22). This is in accordance with previous studies investigating inflammatory markers after remission of CS, including an observational study by Ueland *et al.* which used the same Olink panel for PEA [15], [16], [20], [23]. Since systemic inflammation is involved in the pathogenesis of cardiovascular disease, persistent inflammation could partially explain the reduced quality of life and increased complication and mortality rates in patients that are cured of CS [5], [6], [7], [8].

The main limitation of this study is the small sample size, making analyses of subpopulations and correlations not possible. Ten of the 19 initially included patients dropped out during the postoperative follow-up due to various reasons, indicative of the complicated postoperative period for patients with Cushing’s syndrome. However, even with the limited sample size, we were able to show robust changes in the immune cell phenotype after treatment of CS. Another limitation is that we are not able to distinguish the effects of normalization of glucocorticoid levels and resolution of metabolic syndrome. Likely, both have interfered with the immune cell function. Nonetheless, the findings of this study are of importance for better understanding immunological changes in patients that are cured of CS, although future, larger, studies are needed to confirm these findings and the investigate whether the changes in cytokine production capacity or inflammatory proteome can be used as biomarkers for CS comorbidities.

## Conclusion

In conclusion, after achieving remission of CS, the proinflammatory cytokine production capacity of monocytes increases and anti-inflammatory cytokine production capacity decreases, which could partially explain the higher prevalence of autoinflammatory diseases after remission of CS. Although there are some noticeable changes in the inflammatory proteome, the concentration of the inflammatory markers CRP, IL-8 and IL-6 did not change after remission of CS. Future studies should investigate which mechanisms, like epigenetic or metabolic reprogramming, are involved in the altered immune cell phenotype and whether the phenotype of different (progenitor) immune cell populations in different compartments also changes after achieving remission of CS.

## CRediT authorship contribution statement

**Pepijn van Houten:** . **Annenienke C. van de Ven:** . **Antonius E. van Herwaarden:** Writing – review & editing, Methodology, Investigation. **Mihai G. Netea:** Writing – review & editing, Methodology, Conceptualization. **Martin Jaeger:** Writing – review & editing, Supervision, Project administration, Methodology, Investigation. **Romana T. Netea-Maier:** Writing – review & editing, Supervision, Methodology, Conceptualization.

## Funding

This research did not receive any specific grant from any funding agency in the public, commercial or not-for-profit sector.

## Declaration of competing interest

All authors declare that there is no conflict of interest that could be perceived as prejudicing the impartiality of the research reported.
